# Design of a Sensitive Extracellular Vesicle Detection Method Utilizing a Surface-Functionalized Power-Free Microchip

**DOI:** 10.3390/membranes12070679

**Published:** 2022-06-30

**Authors:** Ryo Ishihara, Asuka Katagiri, Tadaaki Nakajima, Ryo Matsui, Kazuo Hosokawa, Mizuo Maeda, Yasuhiro Tomooka, Akihiko Kikuchi

**Affiliations:** 1 Faculty of Medicine, Juntendo University, Chiba 270-1695, Japan; 2 Department of Materials Science and Technology, Tokyo University of Science, Tokyo 125-8585, Japan; adsm3r@gmail.com (A.K.); ryom.lab@gmail.com (R.M.); kikuchia@rs.tus.ac.jp (A.K.); 3 Department of Bioengineering and Technology, Tokyo University of Science, Tokyo 125-8585, Japan; nakajima.tad.uu@yokohama-cu.ac.jp (T.N.); tomoylab@rs.noda.tus.ac.jp (Y.T.); 4 Bioengineering Laboratory, RIKEN Cluster for Pioneering Research, RIKEN, Saitama 351-0198, Japan; k-hoso@riken.go.jp (K.H.); mizuo@riken.jp (M.M.)

**Keywords:** surface-functionalized power-free microchip, extracellular vesicles, detection sensitivity, point-of-care testing, cancers

## Abstract

Extracellular vesicles (EVs), which are small membrane vesicles secreted from cells into bodily fluids, are promising candidates as biomarkers for various diseases. We propose a simple, highly sensitive method for detecting EVs using a microchip. The limit of detection (LOD) for EVs was improved 29-fold by changing the microchannel structure of the microchip and by optimizing the EV detection protocols. The height of the microchannel was changed from 25 to 8 µm only at the detection region, and the time for EV capture was extended from 5 to 10 min. The LOD was 6.3 × 10^10^ particles/mL, which is lower than the concentration of EVs in the blood. The detection time was 19 min, and the volume of EV solution used was 2.0 µL. These results indicate that an efficient supply of EVs to the detection region is effective in improving the sensitivity of EV detection. The proposed EV detection method is expected to contribute to the establishment of EV-based cancer point-of-care testing.

## 1. Introduction

To treat cancers effectively, it is important to begin treatment before the condition worsens [1,2,3]. However, in the case of cancer, the consultation rates are quite low because current cancer detection methods are time-consuming and expensive. Many cancer biomarkers have been reported to easily detect cancer [4,5,6]. Recently, extracellular vesicles (EVs) present in body fluids have gained popularity as emerging biomarkers in liquid biopsy [7,8,9]; cancers are no exception [10,11,12,13]. It has been reported that the EVs secreted by cancer cells have cancer-specific proteins in their membranes [14]. Therefore, detection of EVs in body fluids can potentially serve as a simple diagnostic method for cancers.

However, existing EV detection methods, such as purification by ultracentrifugation and analysis by immunoblotting or enzyme-linked immunosorbent assay, are cumbersome, time-consuming, and require large sample volumes [15]. To address these problems, point-of-care testing (POCT), a technology that can easily diagnose diseases in clinics or at home, is gaining popularity [16,17]. Examples of its applications include influenza and pregnancy tests. Therefore, to establish a cancer POCT for the improvement of consultation rate and early cancer detection, a device that enables simple, rapid, and highly sensitive EV detection is needed.

Power-free (PF) microchips that utilize the air solubility of poly(dimethylsiloxane) (PDMS) are useful as simple and rapid diagnostic substrates [18]. These chips have the advantages of low cost and short time, and do not require a pump or large sample volume. Previously reported EV detection or isolation methods using microfluidic chips require a pump [19]. In recent years, we have developed a surface-functionalized power-free (SF-PF) microchip [20,21,22,23,24] prepared by functionalizing the inner surface of the microchannel of a PF microchip using radiation-induced graft polymerization. The SF-PF microchip is an excellent platform for POCT devices because it is convenient to add desired functions without compromising the advantages of the PF microchip.

This study aimed to develop a simple and sensitive EV detection method by optimizing the microchannel geometry of the microchip and examining its EV detection protocol to improve the sensitivity of EV detection. We attempted to increase the probability of EVs coming into contact with the detection region by lowering the microchannel height only at that region. This could improve the efficiency as well as the amount of EVs captured at the inner surface detection region of the microchannel. In addition, protocols were investigated to maximize the number of membrane antigens involved in EV capture and optimize the time required to inject EVs into the microchip.

## 2. Materials and Methods

### 2.1. Materials

A PDMS microchip was used as a substrate for UV grafting (Sylgard 184, Dow Corning, Midland, MI, USA). Benzophenone, 2-aminoethyl methacrylate (AEMA), monoclonal mouse anti-CD63 antibody, and FITC-labeled streptavidin were purchased from Sigma-Aldrich (St. Louis, MO, USA). Biotinylated mouse anti-CD63 antibody was purchased from BioLegend (San Diego, CA, USA). Biotinylated rabbit anti-streptavidin (Rockland Immunochemicals, Gilbertsville, PA, USA) and phosphate-buffered saline (PBS, pH 7.4) were purchased from Thermo Fisher Scientific (Waltham, MA, USA). 1-Ethyl-3-(3-dimethylaminopropyl)carbodiimide (EDC) was purchased from FUJIFILM Wako Pure Chemicals Corporation (Osaka, Japan). All other reagents were of analytical grade or higher.

### 2.2. Animals and EV Solution Preparation

The CD1 mice (Sankyo Labo Service Co., Tokyo, Japan) used for serum sampling in this study were maintained under the same conditions as in our previous study [22]. The Institutional Animal Care and Use Committee approved the experimental protocols (approval numbers K16001 and K17008). Male and female mice were anesthetized with isoflurane and sacrificed by decapitation, after which the serum was sampled.

Based on a protocol for the isolation of EVs [25], EVs were isolated from the one-week-cultured conditioned medium of the breast cancer cell line MCF7 by the ultra-centrifugation method. Cell culture and EV isolation conditions were the same as those used in our previous study [22]. After ultracentrifugation, the pellets were resuspended in PBS, and the EV-containing solution was used as a sample solution in the EV-detection experiment. The EVs were freeze-dried and observed by transmission electron microscopy (TEM).

The EV solution (set the protein content to 10 μg) was mixed with sample buffer containing 40 mmol/L dithiothreitol, boiled at 100 °C for 5 min, separated by 8% SDS polyacrylamide gel and transferred onto a PVDF membrane. After blocking with 2% skim milk, the membranes were incubated with each primary antibody, mouse anti-CD63 (1:200; BD Biosciences, Franklin Lakes, NJ, USA) antibody at 4 °C overnight. After incubation with secondary antibody, the proteins were detected using ImmunoStar Zeta (FUJIFILM Wako Pure Chemicals Corporation).

### 2.3. Design and Preparation of a Surface-Functionalized Power-Free (SF-PF) Microchip for Sensitive Extracellular Vesicle (EV) Detection

**Figure 1:**
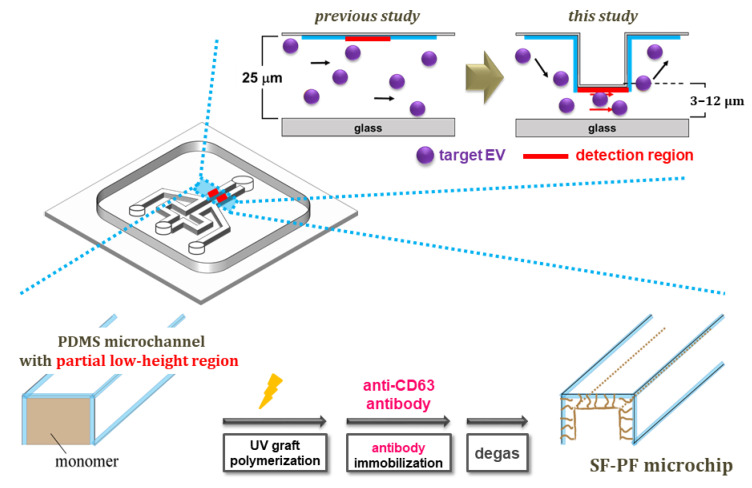
Design of partial low-height microchip channel region and the preparation scheme of a surface-functionalized power-free (SF-PF) microchip for sensitive extracellular vesicle (EV) detection.

A PDMS microchip with a pair of Y-shaped and partial low-height detection regions in the microchannels (total length, width, and height of 2 cm, 100 μm, and 25 μm, respectively) was prepared and evaluated as follows (Figure 1). The lower region was placed 1000 µm away from the cross point of the microchannel (500 μm length and 3–12 μm height). The master mold for the microchip was prepared as follows [26]. To change the partial height of the master mold for the microchannels, as the first layer, a photoresist (SU8-5) was coated by spin coating on an aligned silicon wafer and baked at 90 °C for 1 h. UV light was then irradiated through a photo mask and baked at 90 °C for 30 min. The irradiated wafer was developed using an SU8 developer for 7 min and washed with 2-propanol for 2 min. As the second layer for the partial low-height region of the microchip, a photoresist (SU8-25) was coated on the aligned silicon wafer using a spin coater and baked at 90 °C for 1 h. UV light was then irradiated through a photo mask and baked at 90 °C for 30 min. The irradiated wafer was developed using an SU8 developer for 7 min and washed with 2-propanol for 2 min. Finally, the wafer was hard baked at 140 °C for 10 min. Thus, a partial high-height master mold, which provides partial low-height microchannels of the microchip (500 μm in a 20,000 μm microchannel), was obtained. A PDMS microchip with a pair of Y-shaped and partial low-height microchannel regions was fabricated using the master mold by soft lithography [26]. The silicone elastomer base and silicon elastomer curing agent of the Sylgard 184 kit were mixed at a ratio of 10:1 by weight. The mixture was poured into the master mold and heated at 60 °C for 1 h. The PDMS microchip was then peeled from the master mold and heated at 100 °C for 1 h. The partial lower region of the microchannel (the detection region) was confirmed by observing the fluorescent-labeled DNA (TTTTTT-6FAM) solution-filled microchannel (1 µmol/L in water, 5 µL) using fluorescent microscopy.

The SF-PF microchip was fabricated based on a previous report [22]. First, PAEMA was grafted from the inner surface of the microchannel of the microchip using UV grafting [27,28,29,30,31]. The inner surface of the microchannel was impregnated with benzophenone (used as a photo-initiator) (3.0 μL/microchannel, 10 wt.% in acetone) and the microchannel was filled with AEMA as a monomer solution (3 μL/microchannel, 0.5 mol/L in water). The microchip was then irradiated with UV light (Black-Ray™ UV Lamps B-100 A, 365 nm, 100 W, UVP, USA) for 10 min at a distance of 5 cm.

Next, an EV-capture antibody (anti-CD63 antibody) was immobilized on the grafted polymer chain. The PAEMA-grafted microchannels were filled with 1.0 µL of anti-CD63 antibody-containing solution, a mixture of anti-CD63 antibody (10 µL, 1.0 mg/mL in PBS) and EDC (1.0 µL, 4.5 mg/mL in PBS), and incubated at 37 °C for 2 h with 100% humidity. Finally, the anti-CD63-immobilized microchip was degassed for over 1 h and the SF-PF microchip was obtained. 

### 2.4. Design of EV-Detection Protocol on the SF-PF Microchip

**Figure 2:**
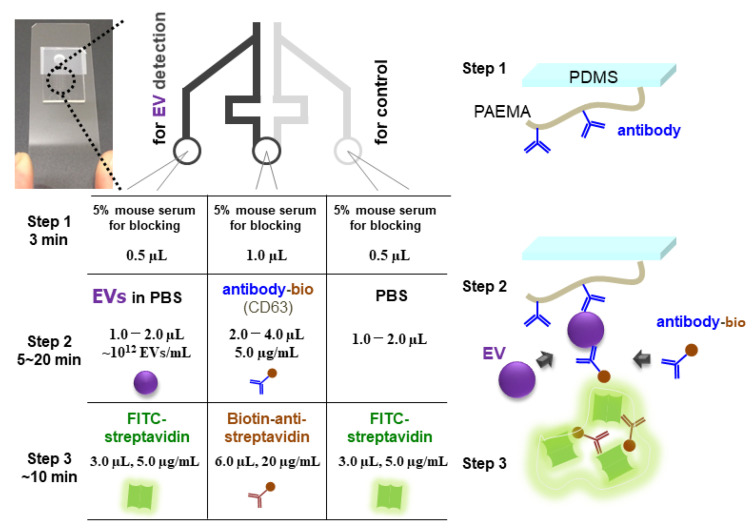
Experimental protocols and schematics of EV detection on the SF-PF microchip.

On the SF-PF microchip with partial low-height microchannel detection regions (3–12 µm height), EVs were detected by laminar flow-assisted dendritic amplification (LFDA) [21,32]. EV detection comprises three steps: Step 1 involves blocking to prevent nonspecific adsorption, Step 2 involves EV capture and sandwich structure formation of immobilized antibody-EV-biotinylated antibody, and Step 3 involves fluorescence amplification by LFDA (Figure 2). For the design of a sensitive EV detection method, in addition to the height of the flow channel, the time required for Step 2 and the separation of Step 2 (EV-capturing and sandwich structure formation steps, Figure S1) were investigated. The detailed experimental conditions are as follows. All injections were conducted by power-free pumping.

In Step 1, 0.5 µL of 5% mouse serum in PBS was injected as a blocking reagent from the left and right inlets, and 1.0 µL was injected from the central inlet for 3 min. In Step 2, 1.0–2.0 µL of EV solution (0–4.6 × 10^12^ particles/mL) was injected from the left inlet, 1.0–2.0 µL of PBS was injected from the right inlet, and 2.0–4.0 µL of biotin-labeled anti-CD63 antibody (0.5–500 µg/mL in PBS) was injected from the central inlet for 5–20 min. In Step 3, fluorescent-labeled streptavidin (F-SA) (5 µg/mL in blocking buffer:1% Roche blocking reagent, 0.02% *w*/*v* sodium dodecyl sulfate, 5× saline sodium citrate, and 0.05% Tween 20) was injected from the left and right inlets, and biotinylated anti-streptavidin antibody (Bio-antiSA) (20 μg/mL in the blocking buffer) was injected from the central inlet to form the fluorescent molecule-containing dendritic structure. Images of the channel were obtained every 1 min under a fluorescence microscope (BZ-8100; Keyence, Osaka, Japan).

### 2.5. Evaluation of EV-Detection Performance of the SF-PF Microchip

The limit of detection (LOD) was calculated using the 3σ criterion. First, the fluorescence intensity of the images was analyzed using an image analysis software (ImageJ 1.45 s, National Institute of Health, Bethesda, MD, USA). Second, the ratio of the fluorescence intensity of the right and left channels (signal-to-blank ratio) was calculated at the time when nonspecific adsorption of the right blank channel started. Based on these data, a calibration curve for EV detection was obtained by fitting using a four parameter logistic function. The LOD was calculated as the EV concentration at the intersection of the calibration curve and 3σ line.

## 3. Results and Discussion

### 3.1. Improvement of EV Detection: Partial Low-Height Microchannel and Optimized Detection Protocols on the SF-PF Microchip

**Figure 3:**
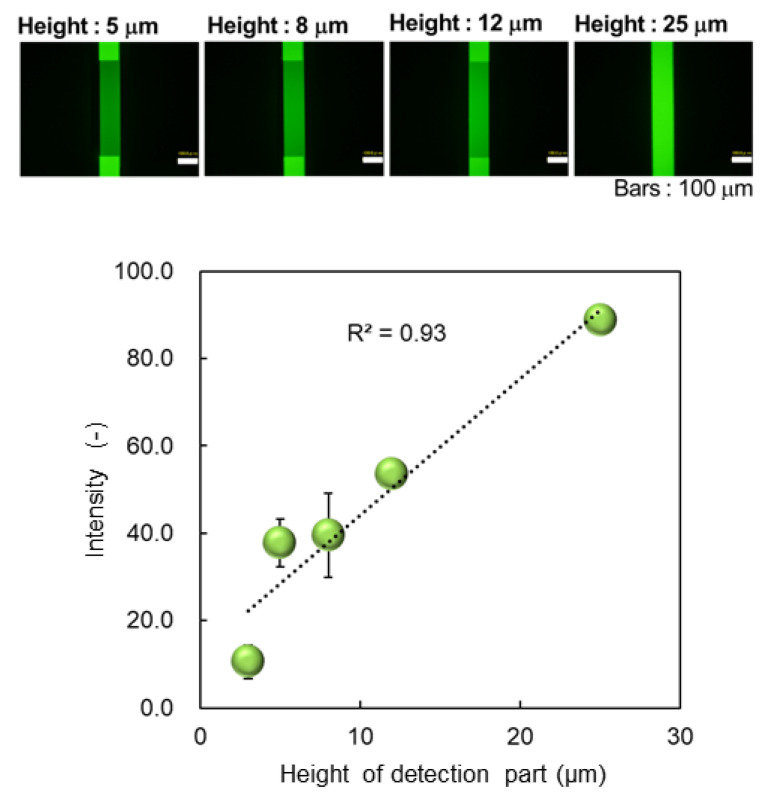
Fluorescent microscope images and fluorescence intensity of poly(dimethylsiloxane) (PDMS) microchannels with different heights only at the detection region filled with fluorescent-labeled DNA (mean ± standard deviation, *n* = 3).

The fluorescence images and fluorescence intensity of PDMS microchannels with different heights only at the detection region filled with fluorescent-labeled DNA are shown in Figure 3. The fluorescence intensity changed only at the detection region of the microchannels of the microchips. The intensity was linear with the expected channel height, confirming the fabrication of the microchip with the height changing only at the detection region.

**Figure 4:**
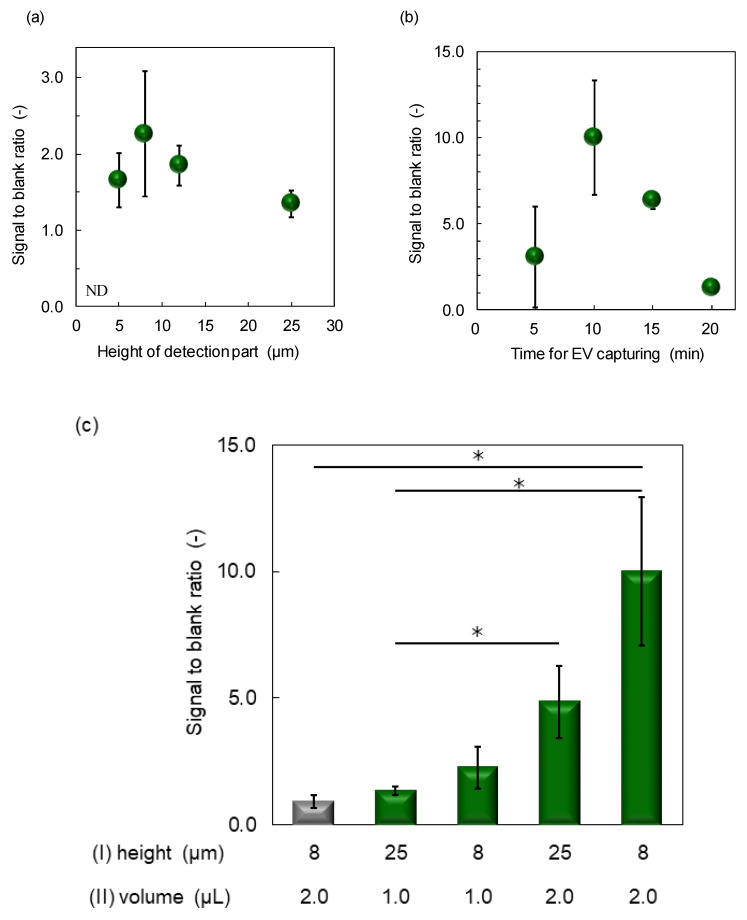
EV detection sensitivity improvement. (**a**) Optimization of height of the detection region of microchannel, (**b**) optimization of time for EV capturing, and (**c**) effect of microchannel height and sample volume on signal-to-blank intensity. * *p* < 0.05.

TEM image of EVs is shown in Figure S2. Expression of CD63 antigen in EVs was confirmed by Western blotting (Figure S3). The signal-to-blank ratio of EV (1.4 × 10^12^ particles/mL), detected using the SF-PF microchip with a different height only at the detection region, is shown in Figure 4a. The signal-to-blank ratio could not be calculated for EV detection using a microchip with a 3 µm channel height at the detection region because the channel was clogged during the experiment. The signal-to-blank ratio increased with decreasing channel height, reaching a maximum at 5–8 µm. This can be attributed to the increase in the contact efficiency between the EV and antibody immobilized on the inner surface of the microchannel as the channel height was partially lowered. To ensure stability of the experiments, we opted to use a channel height of 8 µm only at the detection region in the following experiments.

For the EV-capture step, two approaches were considered. The first for prolonging the EV-capture step (prolonging protocol, Figure 4b), the purpose of which was to capture more EVs and obtain a higher signal intensity. The other step was to separate the EV-capture step (separation protocol, Figure S1). Injection of the EVs and biotinylated antibodies is not simultaneous. EVs were injected first, followed by the biotinylated antibody, to prevent the biotinylated antibody from covering the antigen on the EV membrane, which would eliminate any chances of EVs being captured by the antibody-immobilized inner surface of the microchannels. Considering the possibility of the antigen on the EV membrane being covered, we also investigated the concentration of biotinylated antibody (Figure S4). The prolonging protocol was found to be more effective for the signal-to-blank ratio of EV detection (Figure S1). We also found that the signal-to-blank ratio was not affected by the concentration of biotinylated antibodies (Figure S4). This is because the two solutions are not sufficiently mixed to cover the EV membrane antigens between the confluence and detection region of the microchannel because the flow in the microchannel is laminar and the two solutions are mixed only by diffusion. The large signal increase was attributed to the increased amount of EVs injected into the channel and the increased amount of EVs captured.

Based on these results, we decided to adopt the prolonging protocol and used 5 µL/mL of biotinylated antibody in subsequent experiments. The prolonging protocol was optimal over a time range of 5–20 min (Figure 4b). Power-free pumping is a method in which the flow rate decreases with time and the drive time is approximately 40 min [18]. The limit for the EV-capturing step was 20 min. The signal-to-blank ratio was highest when the EV-capturing time was 10 min, and decreased after 15 min because the subsequent fluorescence amplification was insufficient due to the decrease in flow rate. Based on these results, we concluded that EV supply is the key to improving the sensitivity of EV detection; hence, we decided to extend the EV-capturing time to 10 min in subsequent experiments.

The results of the combination of the two improvements, in which the height of the detection region was lowered to 8 µm and the EV-capture time was extended to 10 min (2 µL) are shown in Figure 4c. The signal-to-blank ratio increased when each of the two improvements was implemented individually, but increased the most (by a factor of five) when the two improvements were implemented in combination.

### 3.2. Sensitive EV Detection Utilizing the Improved SF-PF Microchip and Detection Protocol

**Figure 5:**
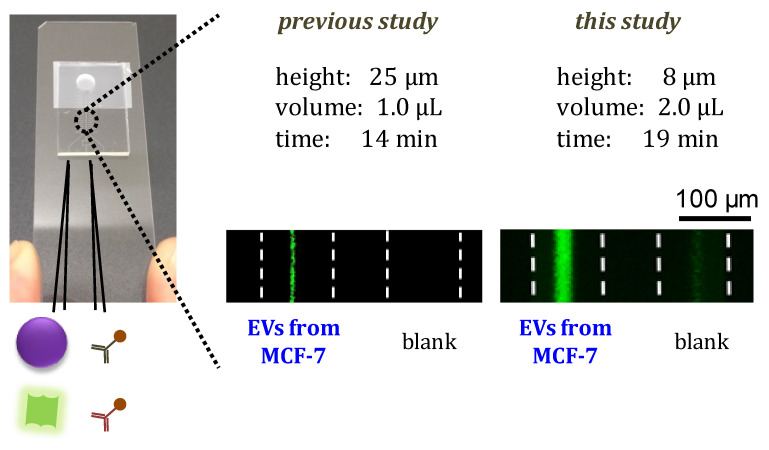
Fluorescent microscope images of EV detection on the SF-PF microchips before and after improvements.

**Figure 6:**
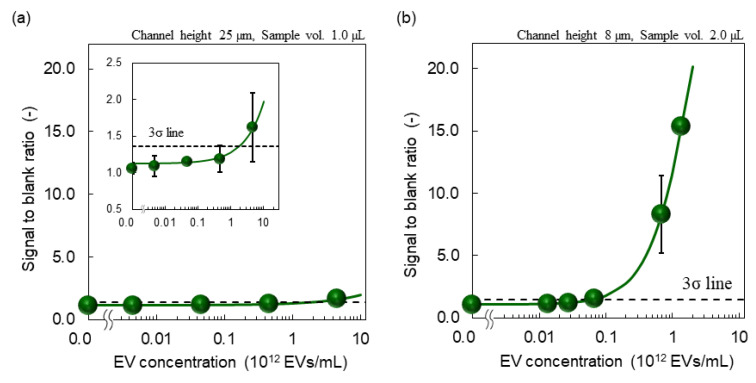
Calibration curves of EV detection on the SF-PF microchips before and after improvements.

A fluorescent microscope image of EV detection using an SF-PF microchip with a constant channel height of 25 µm and EV-capturing time of 5 min (1.0 µL) is shown in Figure 5 and the calibration curve is shown in Figure 6a. A fluorescent microscope image of EV detection using an SF-PF microchip with a height of 8 µm only at the detection region in the microchannel and an extended EV-capturing time of 10 min (2.0 µL) is shown in Figure 5 and the calibration curve is shown in Figure 6b. In both graphs, the signal-to-blank ratio increased as the EV concentration increased. This demonstrates that SF-PF microchips can detect EVs. The LODs were 1.8 × 10^12^ and 6.3 × 10^10^ particles/mL, respectively. These two improvements reduced the LOD by 29-fold, indicating an increase in sensitivity. Since the concentration of EVs in blood is 1.2 × 10^11^–6.0 × 10^11^ particles/mL [33], the proposed method is expected to be able to detect EVs in blood. The detection time in the improved protocol was 19 min, which is 5 min longer than that before the improvement, and the sample volume used was 2.0 µL.

Considering the reported EV detection methods [25,34,35,36,37], the proposed EV detection method on the SF-PF microchip in this study is advantageous with respect to the time and sample volume required for detection. Although some of the reported EV detection methods using microchips [38,39] have femtomolar or attomolar LOD levels, the proposed method is advantageous with respect to device portability. Therefore, these results are sufficient for practical applications. In this study, we focused on the design of a sensitive EV-detection method. The microchannel structure with a partial low-height region and prolonged EV detection protocol were effective for sensitivity improvement. In future studies, the development of fluorescence detection systems [40] and detection by signals other than fluorescence may be necessary to establish a cancer POCT using this microchip and to improve the usability of microchips in the medical field. Additionally, cancer-specific EV detection is necessary to detect cancers. In this case, the microchip preparation method used in this study is only applicable by changing the antibodies. However, further improvement of sensitivity might be necessary because the concentration of cancer-specific EV in the blood is expected to be low. As is also the case with other EV detection methods, this method does not take into account the effect of the size of the EVs being captured and detected. EVs, especially exosomes, are vesicles with a size range of about 30–100 nm, and this method may capture relatively small EVs considering the size-dependent diffusion coefficient. The size distribution of EVs and its influence on detection sensitivity or diagnostic accuracy is an issue for future work.

## 4. Conclusions

We propose a highly sensitive and simple detection method for extracellular vesicles (EVs), which are promising biomarkers for cancer diagnosis. The limit of detection (LOD) was improved 29-fold by optimizing the height of the microchannel at the detection region of the surface-functionalized power-free (SF-PF) microchip and the EV-capturing protocol. The LOD was 6.3 × 10^10^ particles/mL, which is less than the total EV concentration in the blood. The required detection time was 19 min and the EV sample volume was 2.0 µL. These results indicate that the efficient supply of EVs to the detection site is effective in improving the sensitivity of EV detection, and that the EV supply rate is the rate-limiting factor for EV detection. The SF-PF microchip and the detection protocol proposed in this study are expected to serve as a guide for further development of EV detection methods.

To design a microchip that enables highly sensitive detection of EVs, we targeted the membrane antigen CD63, which is commonly found in EVs, especially exosomes, to capture and detect EVs. To establish a cancer point-of-care testing (POCT) using this microchip, it is necessary to specifically detect only EVs secreted by cancer cells in biological samples. For effective treatment, it is desirable to detect the cancer at an early stage. The concentration of EVs secreted by cancer cells was expected to be lower than the total EV concentration in the blood and the LOD in this study. Moreover, the viscosity of biological samples and foreign substances may reduce sensitivity. For these reasons, further improvements in the sensitivity of biological samples are necessary. This was addressed by considering the EV supply rate, which is clarified in this study. Suppression of nonspecific adsorption of EVs until they reach the detection region may contribute to the establishment of EV-based cancer POCT.

## Supplementary Materials

The following supporting information can be downloaded from https://www.mdpi.com/article/10.3390/membranes12070679/s1, Figure S1: Comparison of EV detection protocols; separating and prolonging protocols (mean ± SD, *n* ≥ 3). Figure S2: TEM image of EVs. Figure S3: Expression of CD63 in the EV of MCF10A and MCF7 (*n* = 3). Figure S4: Effect of biotinylated antibody concentration (mean ± SD, *n* ≥ 3).
