# Supplementary material for: Design of a Sensitive Extracellular Vesicle Detection Method Utilizing a Surface-Functionalized Power-Free Microchip
Source: Membranes (Basel). 2022 Jun 30;12(7):679. doi: 10.3390/membranes12070679 (PMC9323264; doi:10.3390/membranes12070679)
Supplement: Supplementary file 1 [file membranes-12-00679-s001.zip › membranes-1728653-supplementary.pdf]

Supporting Information

Design of a Sensitive Extracellular Vesicle Detection Method Utilizing a Surface-Functionalized Power-Free Microchip

Ryo Ishihara [1,*,†], Asuka Katagiri [2,†], Tadaaki Nakajima [3], Ryo Matsui [2], Kazuo Hosokawa [4], Mizuo Maeda [4], Yasuhiro Tomooka [3] and Akihiko Kikuchi [2]

[1] Faculty of Medicine, Juntendo University, Chiba 270-1695, Japan
[2] Department of Materials Science and Technology, Tokyo University of Science, Tokyo 125-8585, Japan; adsm3r@gmail.com (A.K.); ryom.lab@gmail.com (R.M.); kikuchia@rs.tus.ac.jp (A.K.)
[3] Department of Bioengineering and Technology, Tokyo University of Science, Tokyo 125-8585, Japan; nakajima.tad.uu@yokohama-cu.ac.jp (T.N.); tomoylab@rs.noda.tus.ac.jp (Y.T.)
[4] Bioengineering Laboratory, RIKEN Cluster for Pioneering Research, RIKEN, Saitama 351-0198, Japan; k-hoso@riken.go.jp (K.H.); mizuo@riken.jp (M.M.)
* Correspondence: r-ishihara@juntendo.ac.jp
† These authors contributed equally to this work.

Extracellular vesicle (EV) capture step protocol investigation; separating and prolonging protocols

For the EV-capture step, two approaches were considered: one was prolonging the EV-capture step (prolonging protocol, **Fig. 4(b)**), the purpose of which was to capture more EVs and obtain higher signal intensity. The other was separating the EV-capture step (separating protocol, **Fig. S1**). Injection of the EVs and biotinylated-antibody are not simultaneous. First EVs are injected, followed by the biotinylated-antibody. The purpose was to prevent the biotinylated-antibody from covering the antigen on the EV membrane, which would eliminate any chances of the EVs being captured by the antibody immobilized inner surface of the microchannels.

EV (1.4×10^{12} particles/mL) was detected on the SF-PF microchip and the signal to blank ratio was compared. The signal to blank ratio of separating protocol did not increase statistically significantly. The signal to blank ratio of prolonging protocol was increased 4-times (**Fig. S1**). This is because the two solutions are not sufficiently mixed to cover the EV membrane antigens between the confluence and detection region of the microchannel, because the flow in the microchannel is laminar and the two solutions are mixed only by diffusion. The large signal increase was attributed to the increased amount of EVs injected into the channel and the increased amount of EVs captured. Based on these results, we decided to adopt the prolonging protocol in following experiments.

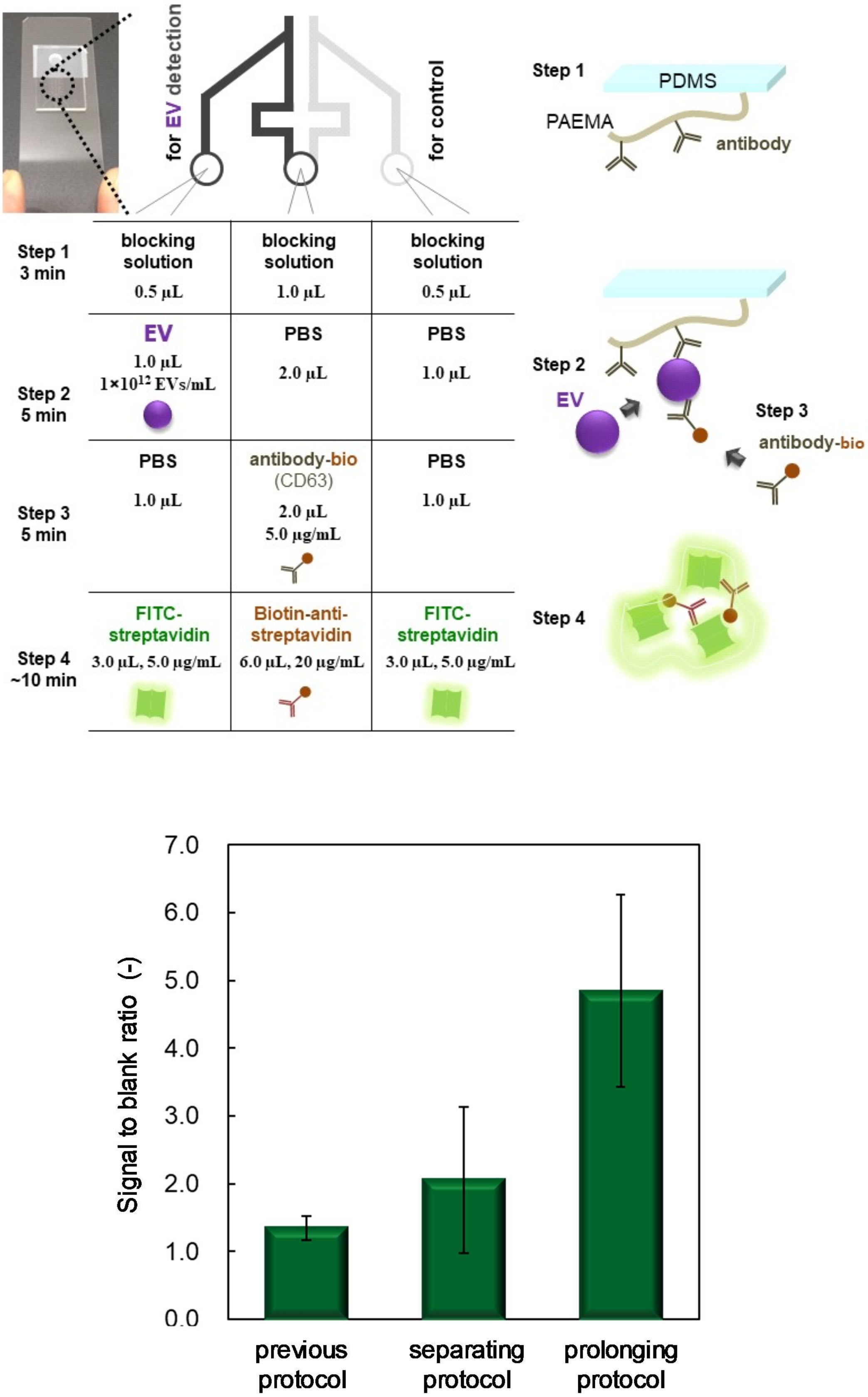

Figure S1. Comparison of EV detection protocols; separating and prolonging protocols (mean ± SD, n ≧ 3).

Transmission electron microscopy (TEM) observation of EVs secreted from MCF7

The EVs isolated from the conditioned medium of the breast cancer cell line MCF7 by ultra-centrifugation method were freeze-dried and observed by TEM (**Fig. S2**)

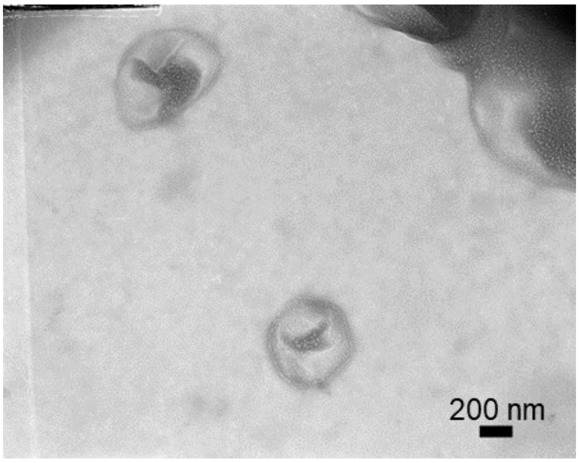

Figure S2. TEM image of EVs.

Detection for CD63 antigen in the EV of MCF7

We confirmed CD63 expression in the EV secreted from MCF7 by Western blotting (**Fig. S3**). MCF10A was used as a normal type of epithelial cell line of mammary glands. The protein expression of CD63 was detected in the EV of MCF10A and MCF7, suggesting that the CD63 was generally expressed in the EV extracted from mammary gland cells. Thus, we used CD63 to capture the EV of MCF7 on the SF-PF microchip.

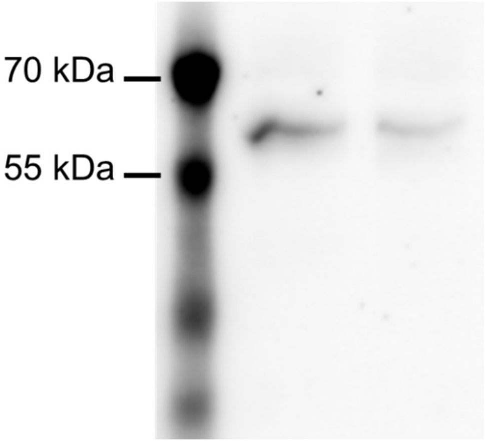

Figure S3. Expression of CD63 in the EV of MCF10A and MCF7 (n = 3).

Optimization of biotinylated antibody concentration

Considering the same possibility of the antigen on the EV membrane being covered as **Fig. S1**, we also investigated the concentration of biotinylated-antibody (**Fig. S4**). It was found that the signal to blank ratio was not affected by the concentration of biotinylated-antibody. This is also because the two solutions are not sufficiently mixed to cover the EV membrane antigens between the confluence and

detection region of the microchannel. Based on these results, we decided to adopt a concentration of 5 μL/mL of biotinylated-antibody in following experiments.

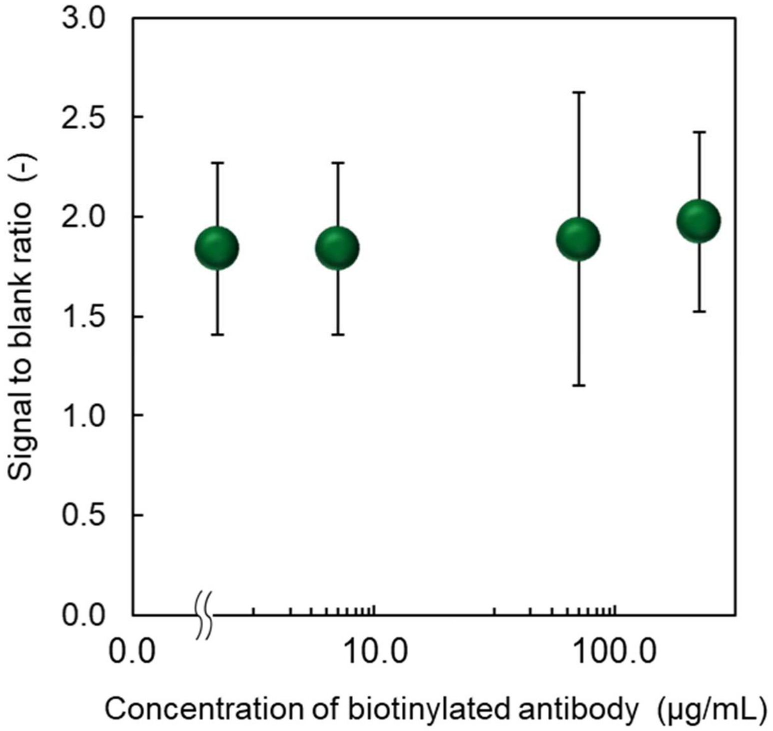

Figure S4. Effect of biotinylated antibody concentration (mean ± SD, n ≧ 3).
